# Social Support Is Protective Against the Effects of Discrimination on Parental Mental Health Outcomes

**DOI:** 10.1177/10783903241243092

**Published:** 2024-04-11

**Authors:** Dallis Alvarez, Harry Adynski, Rebeca Harris, Baiming Zou, Jacquelyn Y. Taylor, Hudson P. Santos

**Affiliations:** 1 Dallis Alvarez, BSN, RN, The University of North Carolina at Chapel Hill, Chapel Hill, NC, USA; 2 Harry Adynski, PhD, RN, PMH-BC, University of California San Francisco, San Francisco, CA, USA; 3 Rebeca Harris BSN, RN, The University of North Carolina at Chapel Hill, Chapel Hill, NC, USA; 4 Baiming Zou, PhD, The University of North Carolina at Chapel Hill, Chapel Hill, NC, USA; 5 Jacquelyn Y. Taylor, PhD, RN, FAHA, FAAN, Columbia University, New York, NY, USA; 6 Hudson P. Santos Jr, PhD, RN, FAAN, University of Miami, Coral Gables, FL, USA

**Keywords:** depression and depressive disorders, stress, pregnancy and postpartum, family support

## Abstract

**BACKGROUND:**

Discrimination, or unfair treatment based on individual characteristics such as gender, race, skin color, and or sexual orientation, is a pervasive social stressor that perpetuates health disparities by limiting social and economic opportunity and is associated with poor mental and physical health outcomes.

**AIMS:**

The purpose of the present study is to (1) examine the association between maternal experiences of discrimination and paternal experiences of discrimination; (2) explore how discrimination relates to parental (maternal and paternal) stress and depressive symptoms; and (3) examine whether social support exerts protective effects.

**METHODS:**

The sample was 2,510 mothers and 1,249 fathers from the Child Community Health Network study. Linear regression models were conducted to explore associations between maternal and paternal discrimination. In addition, mediation analyses were conducted to explore if social support functioned as a mediator between discrimination on parental stress and depressive symptoms.

**RESULTS:**

Most mothers (40.3%) and fathers (50.7%) identified race as the predominant reason for discrimination. Experiencing discrimination was significantly related to stress and depressive symptoms for both parents, and all forms of social support mediated these relationships. Our findings suggest that social support can act as a protective factor against the negative association between discrimination and both stress and depressive symptoms.

**CONCLUSIONS:**

These findings highlight the need to integrate social support into existing interventions and include fathers in mental health screenings in primary-care settings. Finally, we briefly describe the role of nurses and other allied health professionals in addressing discrimination in health care and health policy implications.

Discrimination is a pervasive social stressor that ripples across families, communities, and society. By limiting social and economic opportunities, discrimination carries long-term psychological, physiological, and societal costs (Carter et al., 2017). Discrimination is the unfair treatment of an individual that is perceived as belonging to a certain group and can occur on multiple levels including systemic (e.g., policies, denial of rental opportunity) or individual levels (e.g., microaggressions such as verbal hostility in tone of voice) (Chambers et al., 2021; Jones et al., 2016; Krieger, 2012). Within a family, discrimination can impact individuals on an interpersonal level when discrimination toward one individual in the household (mother/father) can create tension in household relations that affect other family members including children (Condon et al., 2022). Vicarious racism is a specific type of discrimination that results from the indirect experience of hearing about or seeing racist acts against other members of one’s racial group (Chae et al., 2021). In other words, discrimination toward one individual may indirectly affect another, especially within a family (partner or child). These experiences can have far-reaching consequences not only for mothers but for their children (Heard-Garris et al., 2018; Iruka et al., 2022; Saleem et al., 2020). Racial discrimination is one of the most prevalent forms of discrimination in the United States (US) (Gong et al., 2017). Recent studies show that up to 63% of some minoritized groups (studies included samples of Black, Hispanic and Asian individuals) experience racial discrimination in relation to the workplace, health care, housing, education, and or law enforcement (Bergeron et al., 2020; Lee et al., 2019). Discrimination is also linked to poor mental and physical health outcomes including hypertension, obesity, depression, and anxiety (Bergeron et al., 2020; Ibrahim et al., 2021; Kalinowski et al., 2022; Pascoe & Smart Richman, 2009; Santos et al., 2018, 2021).

Chronic exposure to discrimination has been linked to adverse physical and mental health outcomes for parents and their children. Maternally experienced discrimination has been consistently linked to adverse birth outcomes, including pre-term birth, along with the child’s emotional health (Barcelona et al., 2021; Caughy et al., 2004; Collins et al., 2004; Ford et al., 2013; Thayer & Kuzawa, 2015). Discrimination has also been associated with negative effects on emotional wellbeing and may contribute to prenatal and postpartum depression (PPD) through heightened persistent sadness, anhedonia, low energy, changes in eating and sleeping habits, poor concentration, memory, irritability, and suicidal ideation (Stepanikova & Kukla, 2017). Maternal experiences of discrimination and PPD can also increase paternal rates of PPD among African American fathers (Bamishigbin et al., 2017) and impact paternal involvement, relationship outcomes, and the psychological adjustment of other family members (Ansari et al., 2021; Fisher et al., 2021; Kerr et al., 2018). Furthermore, parental PPD is a known risk factor for poor child development, and this risk is higher among families with parents experiencing discrimination (Caughy et al., 2004; Ertel et al., 2012; Santos et al., 2021; Stepanikova & Kukla, 2017; Tran, 2014). Despite the known adverse impacts of discrimination on family health and well-being, there is still a limited understanding of how maternal and paternal experiences of discrimination may be linked to each other and to subsequent mental health outcomes.

Given parent’s vulnerability during the postnatal period, as well as the potential for poor mental health outcomes associated with discrimination, it is vital to identify factors that protect against stress and depressive symptoms (Landy et al., 2012). One potential protective factor is social support (Sherbourne & Stewart, 1991) which may buffer the effects of stressors such as discrimination by providing emotional (the expression of positive affect, empathetic understanding, and the encouragement of expression of feelings) or informational (the offering of advice, information, guidance or feedback) support (Bergeron et al., 2020). Recent studies demonstrate that individuals who face racial discrimination are less likely to experience poor mental health outcomes when they have social support (Bergeron et al., 2020; Brody et al., 2014; Brown et al., 2012; Ickovics et al., 2011; Pao et al., 2019). For example, a study looking at young African American adults in the rural south showed that emotional support may reduce the impact of racial discrimination on biological stress regulation systems (Brody et al., 2014). Another study focused on a diverse sample in NYC found that social support buffered the relationship between racial discrimination and poor mental health (Bergeron et al., 2020; Millender et al., 2024).

In the current study, we build upon extant research to understand the association between discrimination on parental (maternal and paternal) emotional health including both stress and depressive symptoms (Figure 1). The purpose of this study was to (a) examine the association between maternal experiences of discrimination and paternal experiences of discrimination; (b) explore how discrimination relates to parental (maternal and paternal) depressive and stress symptoms (i.e., whether maternal experiences of discrimination relate to maternal depressive and stress symptoms); and (c) examine whether maternally experienced discrimination predicts paternal depression or perceived stress and (i.e., whether paternal experiences of discrimination predict maternal depressive and stress symptoms). Finally, we will examine the extent to which social support protects against the effects of discrimination.

**Figure 1.**
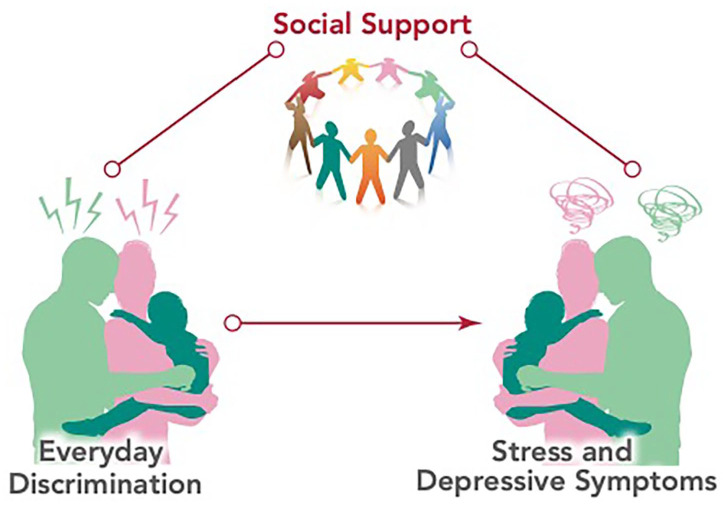
Conceputal Model

## Methods

### Setting

De-identified data from the Community Child Health Network (CCHN) study was used to conduct the present secondary data analysis. The study was approved by The Institutional Review Board of the University of North Carolina at Chapel Hill (#21-2923). The CCHN is an observational study exploring how stressors across multiple levels, such as individual, family, and community factors, may interact with biological factors to contribute to maternal and child health inequalities (Ramey et al., 2015). Multisite recruitment was completed between 2008 and 2010 in North Carolina, Illinois, Maryland, California, and Washington, D.C. While the authors acknowledge not all birthing individuals identify as women CCHN did not collect data on gender identity; therefore, the authors use the term mother, as indicated by the CCHN. The CCHN investigators purposively over-sampled low-income women or women who experienced preterm birth. Mothers were recruited during the peripartum period during either their postpartum hospitalization or during prenatal clinic visits. Women were eligible for enrollment if the following criteria were met: (a) self-identified as Black or African American, Hispanic or Latina, or White; (b) 18 to 40 years old; (c) resided for at least 6-months within data collection locations; (d) had three or fewer children; and (e) had no plans for future sterilization. Fathers were contacted and invited to participate in the study only after mothers gave permission to the researchers (Bamishigbin et al., 2020). Each recruitment site sought approval from the partnering hospital or university on protocols and informed consent procedures.

Recruiters and interviewers consisted of trained personnel and research assistants affiliated with the collaborating academic and community institutions. For this study, we used data from 2,510 mothers and 1,249 fathers from the CCHN study baseline. For additional details about the CCHN study, refer to https://dash.nichd.nih.gov/study/1649.

### Measures

#### Sociodemographic

Sociodemographic data including age, race/ethnicity, education level, and poverty level were included from the baseline visit from both mothers and fathers. Education level was measured as highest level of education received including <high school (HS), HS/Graduation Equivalency Degree (GED), some college, and 4-year degree, and other. Poverty level was based on the Federal Poverty Level (FPL) and was recorded on three levels <100% FPL, 100% to 200% FPL, and >200% FPL.

#### Discrimination

The Everyday Discrimination Scale (EDS) was used to measure parents’ experiences of daily discrimination. This scale includes nine questions that assess perceived discrimination based on routine daily experiences of unjust treatment (Williams et al., 1997). The stem question is: “In your day-to-day life, how often do any of the following things happen to you?” and items include: “You are treated with less courtesy than other people are,” “People act as if they think you are dishonest” and, “You are called names or insulted.” Participants then identify the primary reasons they believe account for these discrimination experiences, such as gender, race, skin color, and/or sexual orientation. This measure is widely used to measure discrimination experiences (Williams et al., 1997). Responses were given on a Likert-type scale with scores range from 0 to 5. Higher scores on the scale indicate greater perceptions of discrimination. Previously the EDS has been validated using the CCHN samples with a reported internal validity of α = .89 (mothers and fathers) (Dunkel Schetter et al., 2013).

#### Social Support

The Medical Outcomes Study Social Support Survey (MOS-SSS) was used to measure parents’ perceived social support. The survey consists of 19 questions that measure four dimensions of social support including emotional support (the expression of positive affect, empathetic understanding and the encouragement of expression of feelings), tangible support (the provision of material aid or behavioral assistance), positive social interaction (the availability of other persons to do fun things with you), and affectionate support (involving expressions of love and affection) (Sherbourne & Stewart, 1991). Each of the 19 items were given on a 5-point Likert-type scale with higher scores indicating greater perceived social support. Within our sample, the internal reliability was strong, with the total score for mothers Cronbach α = 0.96 (all subscales Cronbach α >0.84) and fathers Cronbach α = 0.95 (all subscales Cronbach α >0.80).

### Outcomes

#### Perceived Stress

The Perceived Stress Scale (PSS) was utilized to measure parental stress symptoms (Cohen et al., 1983). The PSS includes 10 items that assess frequency and degree to which individuals have perceived life to be overwhelming, unpredictable, and uncontrollable over the last month. Responses were given on a Likert-type scale of 1 to 5 and a sum score was used with higher scores indicating more perceived stress. Previous studies have demonstrated adequate reliability with Cronbach α ranging from 0.75 to 0.91 (Cohen et al., 1983; Cohen & Janicki-Deverts, 2012; Cole, 1999).

#### Depressive Symptoms

The Edinburgh Postnatal Depression Scale (EPDS) was utilized to measure parental depressive symptoms. The EPDS consists of 10 items that assess depressed mood for the last 7 days. Parents responded based on the frequency of experiencing an item on a 0 to 3 Likert-type scale. The sum of scores ranges from 0 to 30 with higher scores indicating worse depressive symptoms (Cox et al., 1987; McBride et al., 2014).

### Analysis

We conducted descriptive analyses for all demographic variables for the mother and father as well as their stress (PSS) and depressive (EPDS) symptoms at the baseline study visit. In addition, we calculated the means for the discrimination (EDS) as well as the reported frequencies of types of discrimination for both the mother and father. We also provided summary statistics for the Social Support Total Score and subscales (MOS-SSS) for the mother and fathers.

To explore the association between maternal and paternal experiences of discrimination, we conducted statistical analysis using a linear regression model to see if the maternal discrimination total score correlated with the paternal discrimination total score. We controlled for maternal sociodemographic covariates including age, race, education, and poverty factors at a significance level of α < 0.05.

To explore the associations between (a) paternal discrimination on maternal depression; (b) paternal discrimination on maternal stress; (c) maternal discrimination on paternal depression; (d) maternal discrimination on paternal stress; we further conducted regression analysis using four linear regression models by controlling for maternal sociodemographic covariates including age, race, education, and poverty factors. We conducted a mediation analysis of different social supports (M) on the association between discrimination (EDS) and depression (EPDS) by adjusting for age, race, education, and poverty level by adopting the following three multiple linear regression models: The variance estimation for the mediation effect estimate is obtained via 100 rounds of bootstrap resampling with replacement. We report the direct effect, and the parameter estimates for the mediators with accompanying 95% confidence interval, and a *P* value. Similarly, we adopted three multiple linear regression models to investigate different social supports (M) on the association between discrimination (EDS) and stress (PSS) by adjusting for age, race, education, and poverty level. Separate models were conducted for both the maternal and paternal discrimination scores on the two symptom outcomes of stress and depressive symptoms.

## Results

Our sample comprised of 2,510 mothers and 1,249 fathers, respectively. The mean age of mothers was 25.7 years old (*SD* = 5.7), while the mean age of fathers was 29 years old (*SD* = 7.1). The majority of the mothers within the sample self-identified as Black or African American (52.6%) followed by Hispanic (23.7%) and White (21.5%). A similar distribution was present among the fathers with 46.3% of the sample self-identifing as Black or African American, 26.7% Hispanic, and 23.1% White. In the sample, most individuals in both the maternal (42%) and paternal cohort (40.6%) were educated at the HS/GED level. For a full summary of reported demographics, see Table 1.

**Table 1.** Descriptive Statistics for Mothers and Fathers Sociodemographic Data.

Variable	Mothers			Fathers		
	N	% or Mean	*SD*	n	% or Mean	*SD*
**Age**	2,510	25.7	5.7	1,249	29	7.1
**Race/ethnicity**	2,509	–	–	1,735	–	–
African American	1,320	52.6%	–	804	46.3%	–
White/Caucasian	540	21.5%	–	401	23.1%	–
Latina or Hispanic	594	23.7%	–	464	26.7%	–
American Indian or Native American	14	0.6%	–	0	0.0%	–
Asian American or Pacific Islander	0	0	–	14	0.8%	–
Other	78	0.03	–	14	0.8%	–
**Education**	2,608	–	–	1,472	–	–
<HS	481	18.4	–	333	22.6	–
HS/GED	1,095	42	–	597	40.6	–
Some college	568	21.8	–	228	15.5	–
4-year degree	362	13.9	–	224	15.2	–
Other	102	3.9	–	90	6.1	–
**Poverty**	2,510	–	–	1,436	–	–
<100% FPL	1,079	43.0	–	437	30.4	–
100%-200% FPL	688	27.4	–	329	22.9	–
>200% FPL	743	29.6	–	670	46.7	–

*Note.* High school (HS), Graduation Equivalency Degree (GED), Federal Poverty Level (FPL).

Maternal and paternal experiences of discrimination were assessed with the EDS, which asks the participant to report the primary reasons for discriminatory experiences. Most mothers (40.3%) and fathers (50.7%) reported race as the primary reason for discrimination (Table 2). Overall, mothers reported a mean discrimination score of 10.4, while fathers reported a mean discrimination score of 13.3.

**Table 2.** Descriptive Statistics for Mothers and Fathers Outcome Mediators.

Variable	Mothers			Fathers		
	n	Mean	*SD*	n	Mean	*SD*
Depression EPDS	2,406	4.6	4.6	1,216	3.8	3.9
Stress PSS	2,403	12.4	6.6	1,216	10.7	6.1
Social support total score	2,508	83.7	18.1	1,432	85.3	17.2
Emotional support	2,514	83.2	20.0	1,433	82.9	20.1
Tangible support	2,514	81.2	21.4	1,433	84.1	19.5
Affectionate support	2,514	89.8	18.3	1,433	91.2	16.9
Positive social interaction	2,514	83.6	21.4	1,433	88.3	17.8

*Note.* Edinburgh Postnatal Depression Scale (EPDS), Perceived Stress Scale (PSS)

In regard to stress symptoms as measured by the PSS, the maternal average score was 12.4 (*SD* = 6.6) while the paternal average score was 10.7 (*SD* = 6.1) out of 50. With reference to depressive symptoms, as measured by the EPDS, the maternal average was 4.6 (*SD* = 4.6) and the paternal average was 3.8 (*SD* = 3.9) out of 30. For a full report of stress and depressive symptoms, refer to Table 3.

**Table 3.** Perception of Source of Discrimination for Mothers and Fathers Based on the Everyday Discrimination Scale.

Discrimination Source	Ancestry	Gender	Race	Skin Color	Language	Sexual Orientation	Age	Height/Weight	Other
**Mother**									
n	1,691	1,689	1,694	1,690	1,692	1,691	1,693	1,690	1,467
%	13.7	28.8	40.3	19.6	17.4	4	31.7	12.7	48.5
**Father**									
n	979	981	982	981	982	981	1,014	978	537
%	17.1	22.8	50.7	28	19.2	3.2	18.7	12.7	83.2

### Association Between Mothers and Fathers’ Discrimination Experience

The maternal discrimination score and paternal discrimination score were negatively associated. However, this relationship was nonsignificant with a partial correlation of −0.01, 95% CI [−0.06, −0.04], *p* = .770.

### Effects of Paternal Discrimination on Maternal Depression and Maternal Stress

The paternal discrimination score had a negative association with maternal depression (Effect = −0.02, 95% CI [−0.05, 0.01], *p* = .237) and on maternal stress (Effect = −0.03, 95% CI [−0.07, 0.02], *p* = .259). However, both of these relationships were not statistically significant.

### Effects of Maternal Discrimination on Paternal Depression and Paternal Stress

The maternal discrimination score had a positive association with paternal depression (Effect= 0.02, 95% CI [−0.01, 0.05], *p* = .206) and paternal stress symptoms (Effect = 0.04, 95% CI [−0.01, 0.09], *p* = .101). However, both of these associations were not statistically significant.

### Social Support as a Mediator Between Discrimination and Depressive Symptoms

A mediation analysis was conducted to explore whether different forms of social support acted as mediators in the association between maternal discrimination (EDS) and maternal depressive symptoms (EPDS), while adjusting for confounding factors such as age, race, education, and poverty. The total discrimination effect on maternal depressive symptoms is estimated as Effect = 0.20, 95% CI [0.18, 0.22], *p ≤* .001. The total discrimination effect on paternal depressive symptoms is estimated as: Effect = 0.15, 95% CI [0.12, 0.18], *p ≤* .001. In summary, all aspects of social support mediated the effect of discrimination on depressive symptoms for both the mothers and the fathers. Specifically, emotional support served as the strongest mediator for depressive symptoms in both mothers and fathers. The estimated mediation effect of emotional support on depressive symptoms for the mothers is Effect = 0.04, 95% CI [0.029, 0.046], *p ≤* .001. The estimated mediation effect of emotional support on depressive symptoms for the fathers is Effect = 0.03, 95% CI [0.02, 0.05], *p ≤* .001). In addition, the total social support score had a greater mediating effect against depressive symptoms for the mothers when compared to the fathers. The direct effect and mediation effect estimate, corresponding 95% CI estimate, and *P* value under different social support mediators are summarized in Table 4.

**Table 4.** Direct and Indirect Discrimination Effect on Maternal and Paternal Depression Via Different Aspects of Social Support.

Mother						
Direct	95% CI	*p* value	Mediator	Estimate	95% CI	*p* value
0.15	(0.13, 0.17)	<.001	Total	0.05	(0.03, 0.05)	<.001
0.16	(0.14, 0.18)	<.001	Positive social interaction	0.04	(0.02, 0.04)	<.001
0.17	(0.14, 0.19)	<.001	Affectionate support	0.03	(0.02, 0.03)	<.001
0.17	(0.14, 0.19)	<.001	Tangible support	0.03	(0.02, 0.04)	<.001
0.16	(0.14, 0.18)	<.001	Emotional support	0.04	(0.03, 0.04)	<.001
**Father**						
0.11	(0.08, 0.14)	<.001	Total	0.04	(0.02, 0.05)	<.001
0.12	(0.09, 0.15)	<.001	Positive social interaction	0.03	(0.02, 0.04)	<.001
0.12	(0.09, 0.14)	<.001	Affectionate support	0.03	(0.02, 0.04)	<.001
0.12	(0.09, 0.15)	<.001	Tangible support	0.03	(0.02, 0.04)	<.001
0.12	(0.09, 0.14)	<.001	Emotional support	0.03	(0.02, 0.04)	<.001

### Social Support as a Mediator Between Discrimination and Perceived Stress

Similarly, we adopted the above three multiple linear regression models to investigate different maternal and paternal social supports on the association between discrimination and stress symptoms (PSS) by adjusting for age, race, education, and poverty confounding factors. The total discrimination effect on maternal stress was estimated as: Effect = 0.25, 95% CI [0.22, 0.28], *p ≤* .001. The total discrimination effect on paternal stress was estimated as: Effect = 0.24, 95% CI [0.19, 0.28], *p ≤* .001). All forms of social support mediated the effect of discrimination on perceived stress for both the mothers and the fathers, but the effects of total social support and emotional support were more pronounced for the mothers. Specifically, emotional support served as the strongest mediator for perceived stress in both mothers and fathers. The estimated mediation effect of emotional support on perceived stress for the mothers is Effect = 0.06, 95% CI [0.05, 0.08]; *p ≤* .001). The estimated mediation effect of emotional support on perceived stress for the fathers is Estimate = 0.05, 95% CI [0.03, 0.08], *p ≤* .001). The direct effect and mediation effect estimate, corresponding 95% CI estimate, and *p* value under different social support mediators are summarized in Table 5.

**Table 5.** Direct and Indirect Discrimination Effect on Maternal and Paternal Stress Via Different Aspects of Social Support.

Mother						
Direct	95% CI	*p* value	Mediator	Estimate	95% CI	*p* value
0.18	(0.14, 0.21)	<.001	Total	0.07	(0.05, 0.08)	<.001
0.20	(0.16, 0.22)	<.001	Positive social interaction	0.06	(0.04, 0.07)	<.001
0.21	(0.17, 0.24)	<.001	Affectionate support	0.04	(0.03, 0.05)	<.001
0.20	(0.17, 0.23)	<.001	Tangible support	0.05	(0.03, 0.06)	<.001
0.19	(0.15, 0.22)	<.001	Emotional support	0.06	(0.04, 0.07)	<.001
**Father**						
0.18	(0.13, 0.22)	<.001	Total	0.06	(0.03, 0.07)	<.001
0.20	(0.15, 0.24)	<.001	Positive social interaction	0.04	(0.02, 0.05)	<.001
0.20	(0.15, 0.24)	<.001	Affectionate support	0.04	(0.02, 0.05)	<.001
0.20	(0.15, 0.24)	<.001	Tangible support	0.04	(0.02, 0.05)	<.001
0.18	(0.13, 0.22)	<.001	Emotional support	0.05	(0.03, 0.07)	<.001

## Discussion

In this study, we aimed to explore the association between parental experiences of discrimination, the relationship between discrimination, stress, and depressive symptoms, as well as the protective role of social support. The results showed no significant association between maternal and paternal experiences of discrimination. For both mothers and fathers, however, discrimination was associated with stress and depressive symptoms. Those associations were significantly mediated by social support, especially emotional support.

Our first analysis explored the association between maternal and paternal perceived discrimination. Our findings indicated a small negative association (*r* = −0.01) between maternal and paternal experiences of discrimination with and without adjustments for sociodemographic covariates; however, these results were nonsignificant. A previous study has demonstrated an association between maternal and paternal experiences of racial discrimination and prenatal perceived stress (*r* = 2.09), prenatal depression (*r* = 1.85) and postnatal depressive symptoms (*r* = 1.66) (Bécares & Atatoa-Carr, 2016). These results may be related to differences in population characteristics and analytical methods. For example, Bécares & Atatoa’s longitudinal study took place in New Zealand and included a sample of 6822 mothers and 4401 fathers, as opposed to our cross-sectional study that included a sample of 2,510 mothers and 1,249 fathers in the United States. Within this study, there was no association between maternal and paternal discrimination. Nor did we find associations between maternal discrimination and paternal depression or stress and vice versa. We sought to explore if the experience of discrimination between parents influenced depressive and stress symptoms on an interpersonal level (for example vicarious racism), which suggests that the effects of discrimination may extend beyond the intended target. Our findings did not support that the perceived discrimination of one parent had an influence on the other parent’s stress and depressive symptoms within our sample. Nevertheless, the effect of experiences of discrimination between family members should be further investigated to better understand the interdependent effects of discrimination within a family.

We also examined the individual experiences of discrimination and its relation to depressive and stress symptoms. In both mothers and fathers, we found statistically significant positive associations between discrimination, stress, and depressive symptoms. These findings add to the existing literature reflecting how experiences of discrimination are linked to negative impacts on both mental and physical health outcomes (Bergeron et al., 2020; Santos et al., 2018, 2021; Williams et al., 1997). We found that race and skin color were the two primary reasons for both maternal (40.3%, 31.7%) and paternal (50.7%, 28.7%) experiences of discrimination. While studies have examined the effects of maternally experienced discrimination, limited studies have included fathers as well (Bécares & Atatoa-Carr, 2016; Gee et al., 2012). Thus, our study adds to the limited pool of research that explores perceived discrimination and mental health for both mothers and fathers. Our results demonstrate a need for health care–related interventions that protect patients and promote maternal and paternal mental health.

Our final analysis explored the extent to which social support serves as a protective factor between the association of discrimination and both stress and depressive symptoms. Our results show that social support serves as a significant protective factor for both parents. All four types of social support measured including emotional support, tangible support, positive social interaction, and affectionate support protected against the effects of racial discrimination for both mothers and fathers. These results add to existing findings that have established social support as a protective factor against the effects of racial discrimination (Bergeron et al., 2020; Lewis et al., 2015). In addition, we found that for both mothers and fathers, emotional support was estimated to have the greatest mediating effect on both depressive and stress symptoms. Another study found the perceived availability of emotional support, including having someone to confide in, as a mediating factor against the negative mental health effects of perceived discrimination (Ajrouch et al., 2010). Future studies may want to consider exploring the mechanisms by which perceived social support acts as a protective factor. Overall, these results suggest that social support should be incorporated into health care–related interventions and policies that mitigate depressive and stress symptoms related and unrelated to discrimination.

Our results highlight the association between discrimination, elevated stress and depressive symptoms, and the protective role of social support during the perinatal period. Integrating social support into perinatal depression interventions have shown mixed results (Dennis & Dowswell, 2013; Small et al., 2011), and few of these studies incorporate fathers. Fathers who experience PPD indicate their need for social support from formal (health professionals) and informal sources (friends and families) and believe that interventions should include both information on PPD as well as coping strategies for themselves and their partners (Letourneau et al., 2012). To effectively address the psycho-social needs of families, state-based programs like the Help Me Grow initiative can play a vital role. By fostering collaboration across sectors, these programs can connect families with community-based services such as home visiting programs. These services extend support to various aspects of family needs, including parental mental health services, child health care, early care and education, and family support (Miller et al., 2023). Among high-risk groups, home visiting programs with structured social support delivered from health professionals including nurses has been effective in reducing depressive symptoms (Ammerman et al., 2010; Beatson et al., 2021; Molloy et al., 2021). Home visiting interventions often enhance formal structured support via emotional, informational, and instrumental support from the home visitor (nurse, health professional, lay person) (Byrne et al., 2016). Home visiting interventions should also embed teaching participants skills to form and enhance informal social support networks through friends, family, and within their community once the time-limited intervention supports have ended (Hetherington et al., 2020; Marshall et al., 2018). Group therapy modalities are also an evidence-based intervention for PPD, which include direct social support mechanisms. Participants can share and reflect their experiences, including experiences of discrimination, through mutual support among peers with similar experiences (Goodman & Santangelo, 2011). Within this study, emotional support had the strongest mediation effect between experiencing discrimination and depressive symptoms among mothers and fathers and therefore should be enhanced within existing clinical interventions.

Our findings further support integrating mental health screenings in primary care during the peripartum period. Current recommendations from the U.S. Preventive Task Force (2023a, 2023b) indicate that all adults including pregnant and postpartum persons should be screened for depression and anxiety. The American College of Obstetricians and Gynecologists, American Academy of Pediatrics, and the National Association of Pediatric Nurse Practitioners recommend screening in mothers for depression during prenatal and pediatric primary care (Rafferty et al., 2019; Waldrop et al., 2018). Although these recommendations focus on birthing individuals during the perinatal period our findings support recent recommendations to include birthing partners including fathers in routine mental health screening in the primary-care setting (Le et al., 2023; Rao et al., 2020; Walsh et al., 2020). Nurses are often the health care professional administering and or scoring depressive screening forms (Waldrop et al., 2018). Therefore, primary-care nurses need adequate education on how to implement screening procedures and also need evidence-based clinical decision support algorithms that include referral and follow-up care for individuals with positive screens (Waldrop et al., 2018).

Although our sample was community based, our findings highlight the need to address discrimination in health care. In the United States, health care–related discrimination is prevalent, with estimates suggesting that one in five adults experience discrimination (Nong et al., 2020). Furthermore, more than one-third of Black or African American and Latinx adults, along with their family members, have encountered instances of discrimination within the health care system (Nong et al., 2020). Discrimination in health care is a significant concern that must be actively addressed by nurses, allied health care professionals, and hospital systems to deliver high-quality patient care. Nurses play a crucial role in promoting a safe and inclusive health care environment. To combat discrimination, it is essential for nurses to actively listen to patients, understand their unique needs, and advocate for their rights (Altman et al., 2020). By staying informed about cultural responsiveness, implicit biases, and the impact of discrimination on health outcomes, nurses can contribute to fostering an environment where every patient receives fair and compassionate treatment, ultimately improving overall health care quality (Brooks et al., 2022; James & Okoye, 2023).

Our findings reinforce the existing evidence regarding the adverse relationship between discrimination on the parental mental health. Furthermore, they underscore the prevalence of racism as a prominent form of discrimination within our sample. Discrimination and racism in health care and within nursing is recognized by both the American Academy of Nursing and American Psychiatric Nursing Association (Myers, 2022). We recommend that nurses, health care professionals, nursing schools, and health systems implement strategies to address health care–related discrimination including racism. While not within the scope of this article, patients have identified several strategies to reduce discrimination in postpartum care delivery for individuals of color. These strategies involve both individual health care providers and the health care system as a whole (Altman et al., 2020). For individual health care providers, patients suggest spending quality time with patients, building relationships, providing individualized person-centered care, and fostering partnership in decision-making. For the health care system, improving continuity of care, improving provider and racial-ethnic concordance, addressing health system structure issues, and providing provider education including implicit bias training and curriculum integration are recommended (Altman et al., 2020). For a practical guide for nurses seeking to address their own biases and learn about how racial bias is associated with health inequity see James and Okoye (2023). Nursing schools can incorporate the above patient-centered recommendations into their curriculum by highlighting how bias, structural racism, and social determinants of health intersect within their curriculum (Muirhead et al., 2022). Furthermore, schools of nursing can actively recruit and support both faculty and students of color to increase the diversity within the nursing workforce and in so doing increase provider-patient concordance (Adynski et al., 2023; Bravo et al., 2023; Brooks et al., 2022; Green, 2020; Iheduru-Anderson et al., 2021; Johnson et al., 2020; Matthews et al., 2022). Health care organizations can develop patient reporting systems, provide effective implicit bias training, and develop institutional policies to combat racial discrimination in health care and promote culturally responsive care (Bleich et al., 2021; Hagiwara et al., 2020). Our findings underscore the need to confront discrimination and structural racism in the realm of health policy, particularly in the context of perinatal mental health care. Some recommendations that address structural barriers to perinatal mental health include improving health insurance coverage, paid family leave, and investing in communities and nonacute care settings to improve respectful, culturally appropriate care (Bower et al., 2023; Crear-Perry et al., 2021; Foster et al., 2021).

Some limitations should be considered when contextualizing the findings of our study. This is a cross-sectional analysis; thus, causality interpretation should be avoided. A limitation of these data is that they were collected between 2008 and 2010, which may not reflect individuals’ experiences of discrimination today. Furthermore, this does not reflect the increased awareness of systemic racism in health care and police brutality during the COVID-19 pandemic, given the events surrounding the murders of Ahmaud Arbery, George Floyd, and Breonna Taylor and the increased public awareness of the Black Lives Matter movement. Another limitation of our study is the difference in sample sizes between the maternal (*n* = 2,510) and paternal samples (*n* = 1,249). Despite these limitations, the sheer inclusion of fathers in our sample is a strength, given the limited literature regarding paternal experiences of discrimination and historical underrepresentation of fathers in child and family research (Bamishigbin et al., 2017; Bogossian et al., 2019). Furthermore, this study includes the use of a large national data set (CCHN) that is both racially/ethnically and socioeconomically diverse. Exploring social support as a protective factor is an additional strength that could promote the health and well-being of mothers and fathers by integrating social support into clinical practice and policy (Beeber et al., 2022).

## Conclusion

This study contributes to the limited pool of research related to the effects of discrimination on both mothers and fathers. Our analysis demonstrates significant positive associations between discrimination, stress, and depressive symptoms (individually in mothers and fathers) as well as social support as a protective factor (individually for both mothers and fathers) against the effects of discrimination (stress and depressive symptoms). We recommend that clinical interventions should integrate or enhance social support to best address discrimination and parental mental health. Overall, these findings can inform policy and clinical practices that protect the mental and physical well-being of birthing parents and their families.

## References

[bibr1-10783903241243092] Adynski G. I. Bravo L. G. Emmanuel C. J. Ledford A. Ikharo E. Zaragoza S. . . .Woods-Giscombe C. (2023). Barriers and facilitators to recruitment and retention of underrepresented racial and ethnic minoritized students to PhD programs in nursing: A qualitative descriptive study. Nursing Outlook, 71(3), 101962. 10.1016/j.outlook.2023.101962 37003089

[bibr2-10783903241243092] Ajrouch K. J. Reisine S. Lim S. Sohn W. Ismail A. (2010). Perceived everyday discrimination and psychological distress: Does social support matter? Ethnicity & Health, 15(4), 417–434. 10.1080/13557858.2010.484050 20582775 PMC6436554

[bibr3-10783903241243092] Altman M. R. McLemore M. R. Oseguera T. Lyndon A. Franck L. S. (2020). Listening to women: Recommendations from women of color to improve experiences in pregnancy and birth care. Journal of Midwifery & Women’s Health, 65(4), 466–473. 10.1111/jmwh.13102 32558179

[bibr4-10783903241243092] Ammerman R. T. Putnam F. W. Bosse N. R. Teeters A. R. Van Ginkel J. B. (2010). Maternal depression in home visitation: A systematic review. Aggression and Violent Behavior, 15(3), 191–200. 10.1016/j.avb.2009.12.002 20401324 PMC2855144

[bibr5-10783903241243092] Ansari N. S. Shah J. Dennis C. L. Shah P. S. (2021). Risk factors for postpartum depressive symptoms among fathers: A systematic review and meta-analysis. Acta Obstetricia et Gynecologica Scandinavica, 100(7), 1186–1199. 10.1111/aogs.14109 33539548

[bibr6-10783903241243092] Bamishigbin O. N. Dunkel Schetter C. Guardino C. M. Stanton A. L. Schafer P. Shalowitz M. . . .Raju T. (2017). Risk, resilience, and depressive symptoms in low-income African American fathers. Cultural Diversity & Ethnic Minority Psychology, 23(1), 70–80. 10.1037/cdp0000088 27244219 PMC6644062

[bibr7-10783903241243092] Bamishigbin O. N. Jr. Wilson D. K. Abshire D. A. Mejia-Lancheros C. Dunkel Schetter C. (2020). Father involvement in infant parenting in an ethnically diverse community sample: Predicting paternal depressive symptoms. Frontiers in Psychiatry, 11, Article 578688. 10.3389/fpsyt.2020.578688 PMC7538507 33173524

[bibr8-10783903241243092] Barcelona V. Montalvo-Ortiz J. L. Wright M. L. Nagamatsu S. T. Dreisbach C. Crusto C. A. . . .Taylor J. Y. (2021). DNA methylation changes in African American women with a history of preterm birth from the InterGEN study. BMC Genomic Data, 22(1), 1–9. 34482817 10.1186/s12863-021-00988-x PMC8418749

[bibr9-10783903241243092] Beatson R. Molloy C. Perini N. Harrop C. Goldfeld S. (2021). Systematic review: An exploration of core componentry characterizing effective sustained nurse home visiting programs. Journal of Advanced Nursing, 77(6), 2581–2594. 10.1111/jan.14755 33481301

[bibr10-10783903241243092] Bécares L. Atatoa-Carr P. (2016). The association between maternal and partner experienced racial discrimination and prenatal perceived stress, prenatal and postnatal depression: Findings from the growing up in New Zealand cohort study. International Journal for Equity in Health, 15(1), Article 155. 10.1186/s12939-016-0443-4 PMC5034520 27658457

[bibr11-10783903241243092] Beeber L. Ledford A. Gasbarro M. Zeanah P. Knudtson M. Sprinkle S. . . .Leeman J. (2022). Developing a multicomponent implementation strategy for mental health interventions within the nurse-family partnership: An application of the EPIS framework. Journal of Nursing Scholarship, 54(4), 445–452. 10.1111/jnu.12755 34904787

[bibr12-10783903241243092] Bergeron G. Lundy De La Cruz N. Gould L. H. Liu S. Y. Levanon Seligson A. (2020). Association between racial discrimination and health-related quality of life and the impact of social relationships. Quality of Life Research, 29(10), 2793–2805. 10.1007/s11136-020-02525-2 32444931 PMC7242889

[bibr13-10783903241243092] Bleich S. N. Zephyrin L. Blendon R. J. (2021). Addressing racial discrimination in US Health Care Today. JAMA Health Forum, 2(3), Article e210192. 10.1001/jamahealthforum.2021.0192 36218445

[bibr14-10783903241243092] Bogossian A. King G. Lach L. M. Currie M. Nicholas D. McNeill T. Saini M. (2019). (Unpacking) father involvement in the context of childhood neurodisability research: A scoping review. Disability and Rehabilitation, 41(1), 110–124. 10.1080/09638288.2017.1370497 28853312

[bibr15-10783903241243092] Bower K. M. Geller R. J. Jeffers N. McDonald M. Alhusen J. (2023). Experiences of racism and perinatal depression: Findings from the pregnancy risk assessment monitoring system, 2018. Journal of Advanced Nursing, 79(5), 1982–1993. 10.1111/jan.15519 36630188

[bibr16-10783903241243092] Bravo L. G. Ledford A. Adynski G. I. Ikharo E. Emmanuel C. J. Harris L. K. . . .Woods-Giscombe C. (2023). Mental health-related barriers and facilitators to PhD program retention among Underrepresented Racial and Ethnic Minoritized (UREM) nursing students: A qualitative inquiry. Issues in Mental Health Nursing, 44(8), 767–777. 10.1080/01612840.2023.2228893 37450896

[bibr17-10783903241243092] Brody G. H. Lei M. K. Chae D. H. Yu T. Kogan S. M. Beach S. R. H. (2014). Perceived discrimination among African American adolescents and allostatic load: A longitudinal analysis with buffering effects. Child Development, 85(3), 989–1002. 10.1111/cdev.12213 24673162 PMC4019687

[bibr18-10783903241243092] Brooks L. Medina R. Pitts C. J. Dahlem C. H. G. Downes L. Beard K. V. (2022). Building a culturally responsive workforce: Faculty of color in nursing education. The Journal for Nurse Practitioners, 18(5), 575–579.

[bibr19-10783903241243092] Brown J. D. Harris S. K. Woods E. R. Buman M. P. Cox J. E. (2012). Longitudinal study of depressive symptoms and social support in adolescent mothers. Maternal and Child Health Journal, 16(4), 894–901. 10.1007/s10995-011-0814-9 21556696

[bibr20-10783903241243092] Byrne F. Grace R. Tredoux J. Kemp L. (2016). Structured social relationships: A review of volunteer home visiting programs for parents of young children. Australian Health Review, 40(3), 262–269. 10.1071/ah15057 26456798

[bibr21-10783903241243092] Carter R. T. Lau M. Y. Johnson V. Kirkinis K. (2017). Racial discrimination and health outcomes among racial/ethnic minorities: A meta-analytic review. Journal of Multicultural Counseling and Development, 45(4), 232–259. 10.1002/jmcd.12076

[bibr22-10783903241243092] Caughy M. O. B. O’Campo P. J. Muntaner C. (2004). Experiences of racism among African American parents and the mental health of their preschool-aged children. American Journal of Public Health, 94(12), 2118–2124. 10.2105/ajph.94.12.2118 15569963 PMC1448601

[bibr23-10783903241243092] Chae D. H. Yip T. Martz C. D. Chung K. Richeson J. A. Hajat A. . . .LaVeist T. A. (2021). Vicarious racism and vigilance during the COVID-19 pandemic: Mental health implications among Asian and Black Americans. Public Health Reports, 136(4), 508–517. 10.1177/00333549211018675 34034574 PMC8203039

[bibr24-10783903241243092] Chambers B. D. Arega H. A. Arabia S. E. Taylor B. Barron R. G. Gates B. . . .McLemore M. R. (2021). Black women’s perspectives on structural racism across the reproductive lifespan: A conceptual framework for measurement development. Maternal and Child Health Journal, 25(3), 402–413. 10.1007/s10995-020-03074-3 33398713

[bibr25-10783903241243092] Cohen S. Janicki-Deverts D. (2012). Who’s stressed? Distributions of psychological stress in the United States in probability samples from 1983, 2006, and 2009. Journal of Applied Social Psychology, 42(6), 1320–1334. 10.1111/j.1559-1816.2012.00900.x

[bibr26-10783903241243092] Cohen S. Kamarck T. Mermelstein R. (1983). A global measure of perceived stress. Journal of Health and Social Behavior, 24, 385–396. 6668417

[bibr27-10783903241243092] Cole S. R. (1999). Assessment of differential item functioning in the Perceived Stress Scale-10. Journal of Epidemiology and Community Health, 53(5), 319–320. 10396541 10.1136/jech.53.5.319 PMC1756880

[bibr28-10783903241243092] Collins J. W. Jr. David R. J. Handler A. Wall S. Andes S. (2004). Very low birthweight in African American infants: The role of maternal exposure to interpersonal racial discrimination. American Journal of Public Health, 94(12), 2132–2138. 10.2105/ajph.94.12.2132 15569965 PMC1448603

[bibr29-10783903241243092] Condon E. M. Barcelona V. Ibrahim B. B. Crusto C. A. Taylor J. Y. (2022). Racial discrimination, mental health, and parenting among African American mothers of preschool-aged children. Journal of the American Academy of Child & Adolescent Psychiatry, 61(3), 402–412. 34153495 10.1016/j.jaac.2021.05.023 PMC8683578

[bibr30-10783903241243092] Cox J. L. Holden J. M. Sagovsky R. (1987). Detection of postnatal depression: Development of the 10-item Edinburgh Postnatal Depression Scale. The British Journal of Psychiatry, 150(6), 782–786. 10.1192/bjp.150.6.782 3651732

[bibr31-10783903241243092] Crear-Perry J. Correa-de-Araujo R. Lewis Johnson T. McLemore M. R. Neilson E. Wallace M. (2021). Social and Structural Determinants of Health Inequities in Maternal Health. Journal of Women’s Health (2002), 30(2), 230–235. 10.1089/jwh.2020.8882 PMC8020519 33181043

[bibr32-10783903241243092] Dennis C. L. Dowswell T. (2013). Psychosocial and psychological interventions for preventing postpartum depression. Cochrane Database of Systematic Reviews 2, Article CD001134. 10.1002/14651858.CD001134.pub3 PMC11936315 23450532

[bibr33-10783903241243092] Dunkel Schetter C. Schafer P. Lanzi R. G. Clark-Kauffman E. Raju T. N. Hillemeier M. M. Network C. C. H . (2013). Shedding light on the mechanisms underlying health disparities through community participatory methods: The stress pathway. Perspectives on Psychological Science, 8(6), 613–633. 10.1177/1745691613506016 26173227 PMC4505627

[bibr34-10783903241243092] Ertel K. A. James-Todd T. Kleinman K. Krieger N. Gillman M. Wright R. Rich-Edwards J. (2012). Racial discrimination, response to unfair treatment, and depressive symptoms among pregnant black and African American women in the United States. Annals of Epidemiology, 22(12), 840–846. 10.1016/j.annepidem.2012.10.001 23123506 PMC4643652

[bibr35-10783903241243092] Fisher S. D. Cobo J. Figueiredo B. Fletcher R. Garfield C. F. Hanley J. . . .Singley D. B. (2021). Expanding the international conversation with fathers’ mental health: Toward an era of inclusion in perinatal research and practice. Archives of Women’s Mental Health, 24(5), 841–848. 10.1007/s00737-021-01171-y 34431009

[bibr36-10783903241243092] Ford K. R. Hurd N. M. Jagers R. J. Sellers R. M. (2013). Caregiver experiences of discrimination and African American adolescents’ psychological health over time. Child Development, 84(2), 485–499. 10.1111/j.1467-8624.2012.01864.x 23020184

[bibr37-10783903241243092] Foster V. A. Harrison J. M. Williams C. R. Asiodu I. V. Ayala S. Getrouw-Moore J. Davis N. K. Davis W. Mahdi I. K. Nedhari A. Niles P. M. Peprah S. Perritt J. B. McLemore M. R. Mask Jackson F. (2021). Reimagining perinatal mental health: An expansive vision for structural change. Health Affairs, 40(10), 1592–1596. 10.1377/hlthaff.2021.00805 34606355 PMC9107292

[bibr38-10783903241243092] Gee G. C. Walsemann K. M. Brondolo E. (2012). A life course perspective on how racism may be related to health inequities. American Journal of Public Health, 102(5), 967–974. 10.2105/AJPH.2012.300666 22420802 PMC3483932

[bibr39-10783903241243092] Gong F. Xu J. Takeuchi D. T. (2017). Racial and ethnic differences in perceptions of everyday discrimination. Sociology of Race and Ethnicity, 3(4), 506–521. 10.1177/2332649216681587

[bibr40-10783903241243092] Goodman J. H. Santangelo G. (2011). Group treatment for postpartum depression: A systematic review. Archives of Women’s Mental Health, 14(4), 277–293. 10.1007/s00737-011-0225-3 21720793

[bibr41-10783903241243092] Green C. (2020). Equity and diversity in nursing education. Teaching and Learning in Nursing, 15(4), 280–283.

[bibr42-10783903241243092] Hagiwara N. Kron F. W. Scerbo M. W. Watson G. S. (2020). A call for grounding implicit bias training in clinical and translational frameworks. The Lancet, 395(10234), 1457–1460. 10.1016/S0140-6736(20)30846-1 PMC7265967 32359460

[bibr43-10783903241243092] Heard-Garris N. J. Cale M. Camaj L. Hamati M. C. Dominguez T. P. (2018). Transmitting trauma: A systematic review of vicarious racism and child health. Social Science & Medicine, 199, 230–240. 10.1016/j.socscimed.2017.04.018 28456418

[bibr44-10783903241243092] Hetherington E. McDonald S. Williamson T. Tough S. (2020). Trajectories of social support in pregnancy and early postpartum: Findings from the All Our Families cohort. Social Psychiatry and Psychiatric Epidemiology, 55(2), 259–267. 10.1007/s00127-019-01740-8 31256206

[bibr45-10783903241243092] Ibrahim B. B. Barcelona V. Condon E. M. Crusto C. A. Taylor J. Y. (2021). The association between neighborhood social vulnerability and cardiovascular health risk among Black/African American women in the InterGEN study. Nursing Research, 70(5), S3. 10.1097/NNR.0000000000000523 PMC8405545 34074961

[bibr46-10783903241243092] Ickovics J. R. Reed E. Magriples U. Westdahl C. Schindler Rising S. Kershaw T. S. (2011). Effects of group prenatal care on psychosocial risk in pregnancy: Results from a randomised controlled trial. Psychology and Health, 26(2), 235–250. 10.1080/08870446.2011.531577 21318932 PMC3311036

[bibr47-10783903241243092] Iheduru-Anderson K. Shingles R. R. Akanegbu C. (2021). Discourse of race and racism in nursing: An integrative review of literature. Public Health Nursing, 38(1), 115–130. 10.1111/phn.12828 33155328

[bibr48-10783903241243092] Iruka I. U. Gardner-Neblett N. Telfer N. A. Ibekwe-Okafor N. Curenton S. M. Sims J. . . .Neblett E. W. (2022). Effects of racism on child development: Advancing antiracist developmental science. Annual Review of Developmental Psychology, 4, 109–132. 10.1146/annurev-devpsych-121020-031339

[bibr49-10783903241243092] James K. F. Okoye N. (2023). Practical strategies to overcome racial bias in nursing. Nursing for Women’s Health, 27(3), 173–178. 10.1016/j.nwh.2023.02.003 37172614

[bibr50-10783903241243092] Johnson T. M. Bryant A. L. Brooks J. Santos H. Jenerette C. Lynn M. R. Rodgers S. (2020). Utilizing courageous dialogue to support minority and disadvantaged background nursing students. Journal of Professional Nursing, 36(1), 23–27. 10.1016/j.profnurs.2019.06.009 32044048

[bibr51-10783903241243092] Jones K. P. Peddie C. I. Gilrane V. L. King E. B. Gray A. L. (2016). Not so subtle: A meta-analytic investigation of the correlates of subtle and overt discrimination. Journal of Management, 42(6), 1588–1613. 10.1177/0149206313506466

[bibr52-10783903241243092] Kalinowski J. Talbert R. D. Woods B. Langford A. Cole H. Barcelona V. . . .Taylor J. Y. (2022). Police discrimination and depressive symptoms in African American women: The intergenerational impact of genetic and psychological factors on blood pressure study. Health Equity, 6(1), 527–532. 36186618 10.1089/heq.2021.0167 PMC9518801

[bibr53-10783903241243092] Kerr J. Schafer P. Perry A. Orkin J. Vance M. O’Campo P. (2018). The impact of racial discrimination on African American fathers’ intimate relationships. Race and Social Problems, 10(2), 134–144. 10.1007/s12552-018-9227-3

[bibr54-10783903241243092] Krieger N. (2012). Methods for the scientific study of discrimination and health: An ecosocial approach. American Journal of Public Health, 102(5), 936–944. 10.2105/AJPH.2011.300544 22420803 PMC3484783

[bibr55-10783903241243092] Landy C. K. Jack S. M. Wahoush O. Sheehan D. MacMillan H. L. (2012). Mothers’ experiences in the Nurse-Family Partnership program: A qualitative case study. BMC Nursing, 11(1), 1–12. 10.1186/1472-6955-11-15 22953748 PMC3499440

[bibr56-10783903241243092] Le J. Alhusen J. Dreisbach C. (2023). Screening for partner postpartum depression: A systematic review. MCN: The American Journal of Maternal/Child Nursing, 48(3), 142–150. 10.1097/nmc.0000000000000907 36744867

[bibr57-10783903241243092] Lee R. T. Perez A. D. Boykin C. M. Mendoza-Denton R. (2019). On the prevalence of racial discrimination in the United States. PLOS ONE, 14(1), Article e0210698. 10.1371/journal.pone.0210698 PMC6328188 30629706

[bibr58-10783903241243092] Letourneau N. Tryphonopoulos P. D. Duffett-Leger L. Stewart M. Benzies K. Dennis C.-L. Joschko J. (2012). Support intervention needs and preferences of fathers affected by postpartum depression. The Journal of Perinatal & Neonatal Nursing, 26(1), 69–80. 10.1097/JPN.0b013e318241da87 22293644

[bibr59-10783903241243092] Lewis T. T. Cogburn C. D. Williams D. R. (2015). Self-reported experiences of discrimination and health: Scientific advances, ongoing controversies, and emerging issues. Annual Review of Clinical Psychology, 11, 407–440. 10.1146/annurev-clinpsy-032814-112728 PMC5555118 25581238

[bibr60-10783903241243092] Marshall J. Birriel P. C. Baker E. Olson L. Agu N. Estefan L. F. (2018). Widening the scope of social support: The Florida maternal, infant, and early childhood home visiting program. Infant Mental Health Journal, 39(5), 595–607. 10.1002/imhj.21737 30074249

[bibr61-10783903241243092] Matthews A. K. Abboud S. Smith A. U. Smith C. Jeremiah R. Hart A. Weaver T. (2022). Strategies to address structural and institutional barriers to success among students of color in nursing programs. Journal of Professional Nursing, 40, 96–104. 35568466 10.1016/j.profnurs.2022.03.005 PMC8979550

[bibr62-10783903241243092] McBride H. L. Wiens R. M. McDonald M. J. Cox D. W. Chan E. K. (2014). The Edinburgh Postnatal Depression Scale (EPDS): A review of the reported validity evidence. In Zumbo B. Chan E. (Eds.), Validity and validation in social, behavioral, and health sciences (pp. 157–174). Springer. 10.1007/978-3-319-07794-9_9

[bibr63-10783903241243092] Millender E. Harris R. M. Bagneris J. R. Marks L. R. Barcelona V. Wong F. Y. . . .Taylor J. Y. (2024). The cumulative influence of perceived discrimination, stress, and coping responses on symptoms of depression among young African American mothers. Journal of the American Psychiatric Nurses Association, 30(2), 322–332. 35833679 10.1177/10783903221105281 PMC9839894

[bibr64-10783903241243092] Miller M. Passarelli M. Zucker S. Ake W. Martini-Carvell K. Dworkin P. (2023). Help Me Grow: A Model of Targeted Universalism to Advance Equity and Promote the Well-Being of All Children. Social Innovations Journal, 20. Retrieved from https://socialinnovationsjournal.com/index.php/sij/article/view/6595

[bibr65-10783903241243092] Molloy C. Beatson R. Harrop C. Perini N. Goldfeld S. (2021). Systematic review: Effects of sustained nurse home visiting programs for disadvantaged mothers and children. Journal of Advanced Nursing, 77(1), 147–161. 10.1111/jan.14576 33038049

[bibr66-10783903241243092] Muirhead L. Brasher S. Broadnax D. Chandler R. (2022). A framework for evaluating SDOH curriculum integration. Journal of Professional Nursing, 39, 1–9. 35272814 10.1016/j.profnurs.2021.12.004

[bibr67-10783903241243092] Myers C. R. (2022). Racial reckoning: Can nursing organizations and nurses prompt necessary changes? Journal of Psychosocial Nursing and Mental Health Services, 60(11), 3–5. 10.3928/02793695-20221005-01 36317837

[bibr68-10783903241243092] Nong P. Raj M. Creary M. Kardia S. L. Platt J. E. (2020). Patient-reported experiences of discrimination in the US health care system. JAMA Network Open, 3(12), Article e2029650. 10.1001/jamanetworkopen.2020.29650 PMC7739133 33320264

[bibr69-10783903241243092] Pao C. Guintivano J. Santos H. Meltzer-Brody S. (2019). Postpartum depression and social support in a racially and ethnically diverse population of women. Archives of Women’s Mental Health, 22(1), 105–114. 10.1007/s00737-018-0882-6 PMC6800248 29968129

[bibr70-10783903241243092] Pascoe E. A. Smart Richman L. (2009). Perceived discrimination and health: A meta-analytic review. Psychological Bulletin, 135(4), 531–554. 10.1037/a0016059 19586161 PMC2747726

[bibr71-10783903241243092] Rafferty J. Mattson G. Earls M. F. Yogman M. W. (2019). Incorporating recognition and management of perinatal depression into pediatric practice. Pediatrics, 143(1), Article e20183260. 10.1542/peds.2018-3260 30559120

[bibr72-10783903241243092] Ramey S. L. Schafer P. DeClerque J. L. Lanzi R. G. Hobel C. Shalowitz M. . . .Raju T. N. (2015). The preconception stress and resiliency pathways model: A multi-level framework on maternal, paternal, and child health disparities derived by community-based participatory research. Maternal and Child Health Journal, 19(4), 707–719. 10.1007/s10995-014-1581-1 25070734

[bibr73-10783903241243092] Rao W. W. Zhu X. M. Zong Q. Q. Zhang Q. Hall B. J. Ungvari G. S. Xiang Y. T. (2020). Prevalence of prenatal and postpartum depression in fathers: A comprehensive meta-analysis of observational surveys. Journal of Affective Disorders, 263, 491–499. 10.1016/j.jad.2019.10.030 31757623

[bibr74-10783903241243092] Saleem F. T. Anderson R. E. Williams M. (2020). Addressing the “myth” of racial trauma: Developmental and ecological considerations for youth of color. Clinical Child and Family Psychology Review, 23(1), 1–14. 10.1007/s10567-019-00304-1 31641920 PMC8845073

[bibr75-10783903241243092] Santos H. P. Jr. Adynski H. Harris R. Bhattacharya A. Rodriguez A. C. I. Cali R. . . .Murgatroyd C. (2021). Biopsychosocial correlates of psychological distress in Latina mothers. Journal of Affective Disorders, 282, 617–626. 33445084 10.1016/j.jad.2020.12.193 PMC7889736

[bibr76-10783903241243092] Santos H. P. Jr. Nephew B. C. Bhattacharya A. Tan X. Smith L. Alyamani R. A. S. . . .Murgatroyd C. (2018). Discrimination exposure and DNA methylation of stress-related genes in Latina mothers. Psychoneuroendocrinology, 98, 131–138. 30144780 10.1016/j.psyneuen.2018.08.014 PMC6204298

[bibr77-10783903241243092] Sherbourne C. D. Stewart A. L. (1991). The MOS social support survey. Social Science & Medicine, 32(6), 705–714. 10.1016/0277-9536(91)90150-b 2035047

[bibr78-10783903241243092] Small R. Taft A. J. Brown S. J. (2011). The power of social connection and support in improving health: Lessons from social support interventions with childbearing women. BMC Public Health, 11(5), 1–11. 10.1186/1471-2458-11-S5-S4 22168441 PMC3247027

[bibr79-10783903241243092] Stepanikova I. Kukla L. (2017). Is perceived discrimination in pregnancy prospectively linked to postpartum depression? Exploring the role of education. Maternal and Child Health Journal, 21(8), 1669–1677. 10.1007/s10995-016-2259-7 28116534 PMC5515992

[bibr80-10783903241243092] Thayer Z. M. Kuzawa C. W. (2015). Ethnic discrimination predicts poor self-rated health and cortisol in pregnancy: Insights from New Zealand. Social Science & Medicine, 128, 36–42. 10.1016/j.socscimed.2015.01.003 25589034

[bibr81-10783903241243092] Tran A. G. (2014). Family contexts: Parental experiences of discrimination and child mental health. American Journal of Community Psychology, 53(1), 37–46. 10.1007/s10464-013-9607-1 24146093

[bibr82-10783903241243092] U.S. Preventive Task Force. (2023a). Anxiety disorders in adults: Screening. https://www.uspreventiveservicestaskforce.org/uspstf/recommendation/anxiety-adults-screening

[bibr83-10783903241243092] U.S. Preventive Task Force. (2023b). Depression and suicide risk in adults: Screening. https://www.uspreventiveservicestaskforce.org/uspstf/recommendation/screening-depression-suicide-risk-adults

[bibr84-10783903241243092] Waldrop J. Ledford A. Perry L. C. Beeber L. S. (2018). Developing a postpartum depression screening and referral procedure in pediatric primary care. Journal of Pediatric Health Care, 32(3), e67–e73. 10.1016/j.pedhc.2017.11.002 29305113

[bibr85-10783903241243092] Walsh T. B. Davis R. N. Garfield C. (2020). A call to action: Screening fathers for perinatal depression. Pediatrics, 145(1), Article e20191193. 10.1542/peds.2019-1193 31879278

[bibr86-10783903241243092] Williams D. R. Yu Y. Jackson J. S. Anderson N. B. (1997). Racial differences in physical and mental health: Socio-economic status, stress and discrimination. Journal of Health Psychology, 2(3), 335–351. 10.1177/135910539700200305 22013026

